# Identification of novel putative rheumatoid arthritis susceptibility genes via analysis of rare variants

**DOI:** 10.1186/1753-6561-3-s7-s131

**Published:** 2009-12-15

**Authors:** Andrew P Morris, Eleftheria Zeggini, Cecilia M Lindgren

**Affiliations:** 1 Genetic and Genomic Epidemiology Unit, Wellcome Trust Centre for Human Genetics, University of Oxford, Roosevelt Drive, Oxford OX3 7BN, UK; 2 Wellcome Trust Sanger Institute, The Morgan Building, Wellcome Trust Genome Campus, Hinxton, Cambridge CB10 1HH, UK

## Abstract

Established loci for rheumatoid arthritis (RA), including *HLA-DRB1 *and *PTPN22*, do not fully account for the genetic component of susceptibility to the disease. One possible source of as yet undiscovered susceptibility genes are those mediated through effects of rare variants. We present a novel method for gene-based genome-wide scans of whole-genome association (WGA) data to identify accumulations of rare variants associated with disease. We apply our method to WGA SNP genotype data obtained from 868 RA cases and 1194 controls. Our results highlight novel putative RA susceptibility genes that have not previously been identified in large-scale WGA studies.

## Introduction

The genetic contribution to susceptibility to rheumatoid arthritis (RA) is well established, with a sibling risk ratio of the order of 5 to 10 [1]. The prevalence of the disease in Caucasians is ~0.8%, the risk being three times higher in females than males [2]. The most recognized RA associations are with haplotypes of the *HLA-DRB1 *locus and variation in *PTPN22*, both of which have been widely replicated [3-5]. However, these two associations explain only half of the familial aggregation of the disease. More recently, large-scale whole-genomic association (WGA) studies have suggested a number of novel RA susceptibility loci including variation close to both the alpha and beta chains of the IL2 receptor (*IL2RA *and *IL2RB*), a single-nucleotide polymorphism (SNP) in linkage disequilibrium with variation at the *TRAF1-C5 *locus, two independent signals on chromosome 6q23, and a female-specific effect on chromosome 7q32 [6-8]. However, despite these successes, a large proportion of the genetic component of RA susceptibility remains unexplained.

Large-scale WGA studies of thousands of individuals are well powered to detect the modest genetic effects we expect for complex diseases, provided that the functional variants are common (minor allele frequency (MAF) greater than 1-5%, depending on the sample size of the study). Although a number of common variants for complex diseases have now been identified and replicated, it seems unlikely that the common-disease, common-variant hypothesis is all encompassing. Instead, some proportion of genetic susceptibility to complex diseases may be attributed to rare variants, potentially acting together within the same functional unit, with a modest joint effect. Such associations cannot be identified through traditional single-SNP analyses because rare variants are poorly captured by WGA genotyping products [9], and are thus under-powered without sample sizes of tens of thousands of individuals which may be practically or financially infeasible.

Here, we used SNP genotype data obtained from 868 RA cases and 1194 unaffected controls of Northern European ancestry using the Illumina HumanHap 550 k BeadChip to identify genes containing accumulations of rare variants associated with disease susceptibility. The log-odds of disease were modeled as a linear function of the proportion of rare SNPs within a gene at which individuals carry at least one copy of the minor allele within a logistic regression framework. Gene-based genome-wide scans for association with RA were performed, fully accounting for the underlying population structure, both with and without adjustment for the effects of the *HLA-DRB1 *locus, and allowing for sex-differentiated effects.

## Methods

Consider a sample of unrelated cases and controls typed for SNPs in a gene or small genomic region. Let *n*_*i *_denote the number of rare SNPs (as defined by some predetermined MAF threshold) for which the *i*^th ^individual has been successfully genotyped, and let *r*_*i *_denote the number of these SNPs at which they carry at least one copy of the rare variant. We can model the log-odds of disease of the *i*^th ^individual in a logistic regression framework, given by

In this expression, **x**_*i *_denotes a vector of covariate measurements for the *i*^th ^individual, with corresponding regression coefficients **β**. The parameter *λ *is the log-odds ratio for an individual carrying a full complement of rare variants compared with an individual carrying none. Thus, we construct a likelihood-ratio test of association of an accumulation of rare variants with disease by comparing the maximized likelihoods of two models via analysis of deviance: i) the null model where *λ *= 0; and ii) the alternative model where *λ *is unconstrained. The contribution of the *i*^th ^individual to the likelihood is weighted by *n*_*i *_to allow for differential call rates between samples.

## Results

Genotypes were reported for 544,892 autosomal and X-chromosome SNPs. A total of 35,760 SNPs were excluded from analysis through application of quality control (QC) filters of low call rate (<97%) and extreme deviation from Hardy-Weinberg equilibrium (study-wide exact *p *< 5.7 × 10^-7^, except in females only for X-chromosome SNPs). To account for population structure, identity-by-state metrics were calculated for each pair of individuals for every fifth SNP passing QC filters with study-wide MAF greater than 1%, to minimize the effects of linkage disequilibrium, and excluding the MHC to eliminate any bias due to the effect of *HLA-DRB1*. Application of multi-dimensional scaling techniques to the resulting matrix of pair-wise identity-by-state statistics generated five axes of genetic variation associated with RA (*p *< 0.001) after adjustment for sex as a covariate. Genes and their boundaries were determined from the UCSC known genes database (build 35), extended by 50 kb up- and down-stream to incorporate additional functional elements and the promoter region. Each gene was considered independently, irrespective of any overlap in their boundaries. A total of 29,073 rare variants, defined to have a study-wide MAF of less than 5%, were assigned to at least one of 25,501 genes, and thus taken forward for analysis.

**Figure 1:**
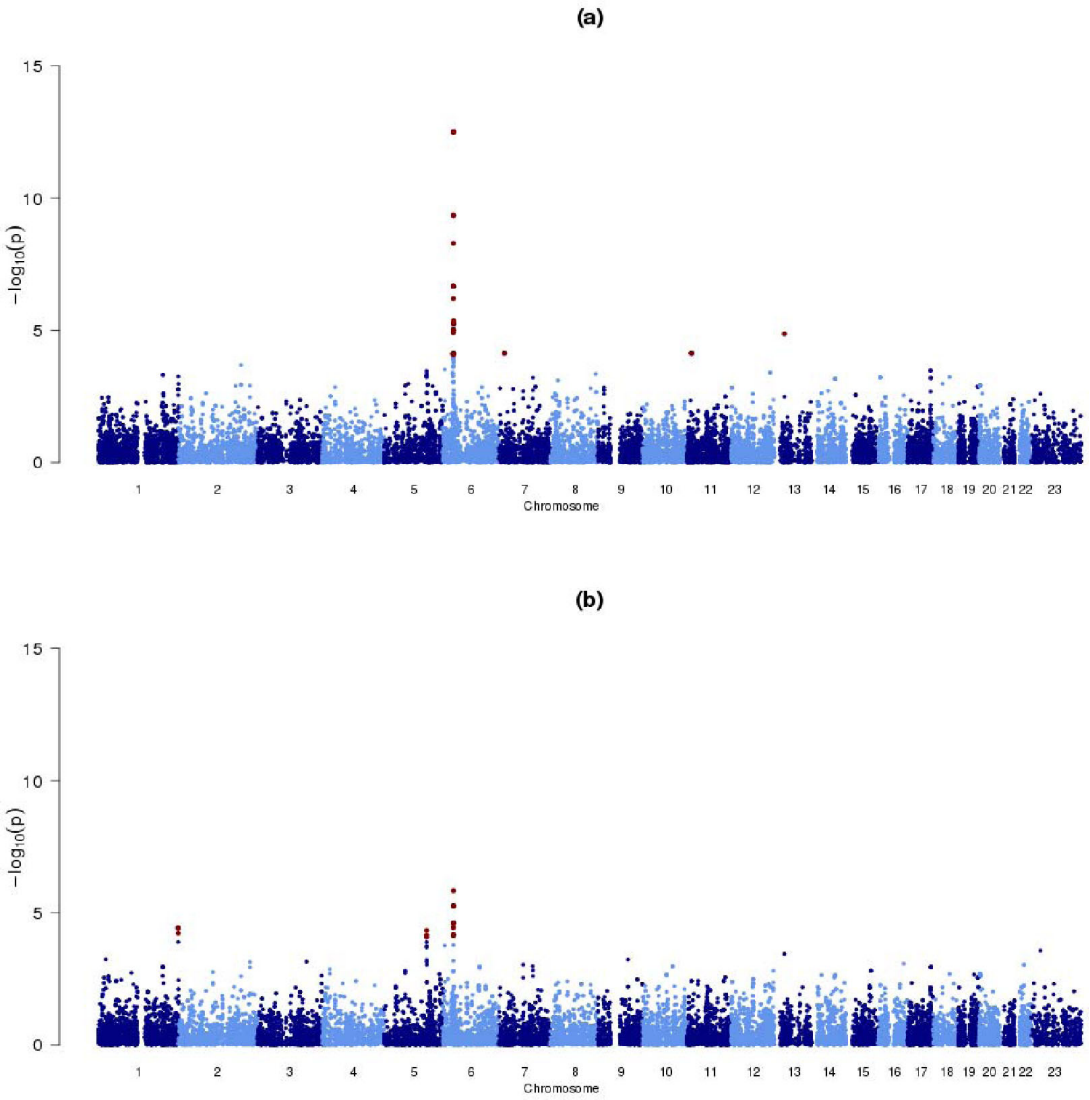
**Summary of gene-based genome-wide scans of association of RA with accumulations of rare variants**. Each gene-based test has been adjusted for: (a) sex and five axes of genetic variation; (b) sex, five axes of genetic variation, and the number of shared epitope alleles to account for the effects of *HLA-DRB1*. Genes achieving a nominal significance threshold of *p *< 10^-4 ^are highlighted in red.

Figure 1a presents results of a gene-based genome-wide scan of association of RA with accumulations of rare variants, adjusted for sex and axes of genetic variation as covariates in the logistic regression model, as a function of gene location. Unsurprisingly, the strongest signals of association were observed for accumulations of rare variants in genes in the major histocompatibility complex (MHC), which are likely to be in linkage disequilibrium with *HLA-DRB1 *risk haplotypes. The strongest signals of association outside of the MHC (*p *< 10^-4^) occurred with *FRY *(furry homolog), *PRPS1L1 *(phosphoribosyl pyrophosphate synthetase 1-like 1), and *ARNTL *(ARNT-like protein 1, brain and muscle), each containing accumulations of rare variants that appear to be protective for RA (Table 1).

**Table 1:** Strongest signals of rare variant association with RA (*p *< 10^-4^) outside of the MHC^a^

		Rare variants			
				
Gene	Chromosome	Number	Mean MAF (%)	Odds ratio (95% CI)	*p*-Value
*FRY*	13	5	2.6	0.10 (0.03-0.28)	1.4 × 10^-5^
*PRPS1L1*	7	1	4.4	0.46 (0.31-0.68)	7.3 × 10^-5^
*ARNTL1*	11	1	3.0	0.38 (0.24-0.61)	7.3 × 10^-5^

^a^Each gene-based test has been adjusted for sex and five axes of genetic variation to account for population structure.

To account for the effects of the *HLA-DRB1 *locus, we repeated our analysis by adjusting for the number of shared epitope alleles, in addition to sex and axes of genetic variation, as covariates in the logistic regression model, the results of which are presented in Figure 1b. The strongest signal of association (*p *= 5.4 × 10^-6^) occurred within the class III region of the MHC, in two overlapping genes, *PBX2 *(pre-B-cell leukemia homeobox 2) and *NOTCH4 *(Notch homolog 4). These two genes contained the same three rare variants, jointly occurring with an odds ratio of 0.08 (95% confidence interval 0.03-0.24) for an individual carrying a minor allele at all of these variants, relative to one carrying none, suggesting them to be protective for RA. This result would indicate RA association with rare variants within the MHC, independent of the effect of *HLA-DRB1*. Outside of the MHC, the strongest signals of association with RA (*p *< 10^-4^) occurred with *TRIM58 *(tripartite motif-containing 58) and *HINT1 *(histidine triad nucleotide binding protein 1), again containing accumulations of rare variants that appear to be protective for RA (Table 2).

**Table 2:** Strongest signals of rare variant association with RA (*p *< 10^-4^) outside of the MHC, adjusting for the effects of HLA-DRB1^a^

		Rare variants			
				
Gene	Chromosome	Number	Mean MAF (%)	Odds ratio (95% CI)	*p*-value
*TRIM58*	1	3	1.1	0.04 (0.01-0.18)	3.7 × 10^-5^
*HINT1*	5	4	3.0	0.25 (0.13-0.48)	4.6 × 10^-5^

^a^Each gene-based test has been adjusted for sex, five axes of genetic variation to account for population structure, and the number of shared epitope alleles to allow for the effects of *HLA-DRB1*.

To test for sex-specific effects, we have performed gene-based genome-wide scans of association with RA within males and females separately, with and without adjustments for the effects of the *HLA-DRB1 *locus. Sex-differentiated tests of association were performed by summing the deviances obtained across the two sexes, adjusted for axes of genetic variation. The strongest signal of association, not identified in the primary analyses described above, occurred in *MYO1B *(myosin 1B), containing six rare variants (*p *= 7.8 × 10^-5 ^without adjustment for the effects of *HLA-DRB1*). There is some evidence of heterogeneity of the effects of accumulations of rare variants in the gene between the sexes (*p *= 0.074). There is a signal of association with RA in females (*p *= 1.0 × 10^-4^), but no such effect is seen in males (*p *= 0.45), although this may reflect a lack of power due to lower sample size.

## Discussion

We have presented the results of novel methodology for gene-based genome-wide scans for association of RA with an accumulation of rare variants. The strongest signals of association were observed for genes in the MHC, where an accumulation of rare variants reduces risk of RA, presumably as a result of linkage disequilibrium with *HLA-DRB1 *haplotypes. There are no rare variants (MAF<5%) on the Illumina HumanHap 550 k beadchip passing our QC filters in *PTPN22*. Rare variants are represented in the other established RA loci (*TRAF1*-*C5*, *IL2RA*, and *IL2RB*), but our results do not indicate any evidence of rare variant effects within these genes.

Our results highlight rare variant associations with RA within the MHC, independent of the effects of the *HLA-DRB1 *locus. Furthermore, a number of novel putative RA susceptibility genes have been identified outside of the MHC (*p *< 10^-4^), with signals of association at least as strong as would be observed through application of traditional single-SNP methods. It is interesting to note that for all of these genes, accumulations of rare variants are associated with *decreased *risk of RA, suggesting them to be protective. This could reflect the fact that we are able to identify more rare variants in the larger sample of controls than cases, and hence that our analysis has greater power to detect protective associations. We repeated our analyses using rare variant thresholds of MAF less than 2% and less than 1%. These analyses highlighted no novel susceptibility genes for RA (*p *< 10^-4^), reflecting the scarcity of variants with MAF less than 2% on the Illumina HumanHap 550 k Beadchip (just 15,093 SNPs covering 18,447 genes).

Our analyses highlighted associations with a just a single rare variant in each of the genes *PRPS1L1 *and *ARNTL1*. Application of traditional single-SNP analysis would thus produce a similar signal of association to that identified here. However, the advantage of a gene-based analysis is that there is a lesser burden of multiple testing, so that a less stringent genome-wide significance level would be sufficient. The definition of such a threshold is difficult here because each gene is treated as independent, despite the fact that many overlap. The results of overlapping genes will be strongly correlated because they share SNPs in common, and thus a simple Bonferroni correction for the number of tests performed will be highly conservative. Genotypes are more difficult to call at rare variants than more common SNPs, the distinction between heterozygotes and rare homozygotes being less transparent. The QC filters implemented here will exclude the worst offenders, but careful visual inspection of the cluster plots of the remaining variants in associated genes is essential. These two issues highlight the importance of confirmation of these findings in independent replication cohorts, or through meta-analysis with other RA WGA studies, such as that carried out as part of the Wellcome Trust Case Control Consortium [7]. Ideally, studies will be performed in the same population because we expect the spectrum of rare variants to be more variable that common SNPs between even closely related populations. In addition, it would be preferable for samples to be typed using the same technology because different panels of rare variants appear on different genotyping products, and are more difficult to impute across platforms than common SNPs [9].

Analysis of rare variants on WGA genotyping arrays is far from ideal, given their scarcity and poor coverage by common SNPs. However, with the increasing availability of large-scale re-sequencing data, such as that generated by the 1,000 Genomes Project [10], we are entering an exciting period for rare variant discovery in which development of analytical strategies to maximize the potential of these investments will be of critical importance.

## Conclusion

We have presented the results of an application of a new method for gene-based genome-wide association of RA with accumulations of rare variants (MAF<5%). Our results confirm strong signals of association for genes in the MHC, and highlight putative novel RA associations which require follow-up in independent replication cohorts from the same population.

## List of abbreviations used

MAF: Minor allele frequency; MHC: Major histocompatability complex; QC: Quality control; RA: Rheumatoid arthritis; SNP: Single-nucleotide polymorphism; WGA: Whole-genome association.

## Competing interests

The authors declare that they have no competing interests.

## Authors' contributions

APM performed the statistical analysis and drafted the manuscript. All authors conceived of the study and read and approved the final manuscript.
